# Raman spectroscopy identifies radiation response in human non-small cell lung cancer xenografts

**DOI:** 10.1038/srep21006

**Published:** 2016-02-17

**Authors:** Samantha J. Harder, Martin Isabelle, Lindsay DeVorkin, Julian Smazynski, Wayne Beckham, Alexandre G. Brolo, Julian J. Lum, Andrew Jirasek

**Affiliations:** 1 University of Victoria, Department of Physics and Astronomy, PO Box 1700 STN CSC, Victoria, British Columbia, V8W 2Y2, Canada; 2 BC Cancer Agency—Vancouver Island Centre, Trev and Joyce Deeley Research Centre, 2410 Lee Ave., Victoria, British Columbia, V8R 6V5, Canada; 3 BC Cancer Agency—Vancouver Island Centre, Medical Physics, 2410 Lee Ave., Victoria, British Columbia, V8R 6V5, Canada; 4 University of Victoria, Department of Chemistry, PO Box 3065, Victoria, British Columbia, V8W 3V6, Canada; 5 University of Victoria, Department of Biochemistry and Microbiology, PO Box 1700 STN CSC, Victoria, British Columbia, V8W 2Y2, Canada; 6 Mathematics, Statistics, Physics, and Computer Science, University of British Columbia Okanagan, 3333 University Way, Kelowna, British Columbia, V1V 1V7, Canada

## Abstract

External beam radiation therapy is a standard form of treatment for numerous cancers. Despite this, there are no approved methods to account for patient specific radiation sensitivity. In this report, Raman spectroscopy (RS) was used to identify radiation-induced biochemical changes in human non-small cell lung cancer xenografts. Chemometric analysis revealed unique radiation-related Raman signatures that were specific to nucleic acid, lipid, protein and carbohydrate spectral features. Among these changes was a dramatic shift in the accumulation of glycogen spectral bands for doses of 5 or 15 Gy when compared to unirradiated tumours. When spatial mapping was applied in this analysis there was considerable variability as we found substantial intra- and inter-tumour heterogeneity in the distribution of glycogen and other RS spectral features. Collectively, these data provide unique insight into the biochemical response of tumours, irradiated *in vivo*, and demonstrate the utility of RS for detecting distinct radiobiological responses in human tumour xenografts.

Lung cancer is the second most common cancer in both men and women, accounting for 13% of all new cancers and 27% of all cancer deaths in Canada^1^. Surgical resection may be an option for early stage non-small cell lung cancer (NSCLC) and, in some cases, post-operative radiation therapy (RT) may be indicated. However, lung cancers in more advanced stages may not be candidates for surgery because of limiting factors such as tumour size, location, lymphatic involvement and the patient’s overall health status. In these cases, definitive RT may be used as an alternative treatment option. Radiation therapy uses high energy ionizing radiation to destroy diseased tissues while minimizing damage to healthy tissues. For NSCLC, typical definitive RT total doses are on the order of 60 Gy, delivered in 2 Gy fractions^2^. Patients with locally advanced NSCLC undergoing definitive RT showed one year survival rates of 18% in a randomized control trial evaluating the role of RT compared to chemotherapy or observation^3^,^4^. Those patients who do not respond successfully to definitive RT may benefit from pre-screening techniques to assess radiation resistance or sensitivity, allowing further guidance in selecting an appropriate treatment regimen for the individual.

Paramount to successful RT treatment is the degree of radiosensitivity or radioresistance associated with a specific patient’s disease. Several factors can influence the extent of tumour response to radiation and, hence, successful outcome of RT including intrinsic radiation response of the tumour cells and the surrounding tumour microenvironment^5^,^6^. As a consequence, there is currently no accepted technique to predict patient radiosensitivity or radioresistance. The ability to determine tissue radioresponse for a given patient would be a major step towards improving treatment outcome in radiotherapy. Predictors of cellular radiation response including deoxyribonucleic acid (DNA) break repair^7^, chromosome aberrations^8^, apoptosis^9^, hypoxia^10^,^11^,^12^, cancer stem cell content^13^ and genetic biomarkers of radioresistance^14^,^15^ have been previously investigated. These techniques can elucidate some of the important factors involved in resistance to radiotherapy in tumours. However, none of these techniques have been adopted in the clinic; presumably because many of them suffer from considerable limitations for clinical implementation, including tumour type specificity, invasiveness (requiring biopsy of tumour tissue) as well as cost in time and labour.

Raman spectroscopy (RS) is a minimally invasive optical technique with the potential for pre-treatment characterization and early monitoring of biomolecular changes related to radiation response within tumour cells and tissues^16^,^17^,^18^,^19^,^20^,^21^,^22^, providing opportunities for personalizing RT treatments. Raman spectra of biological materials in the 400–1800 cm^−1^ range yield a fingerprint of the sample’s biomolecular content. Raman spectroscopy can therefore be applied to detect cellular changes resulting from metabolic processes and offers the potential to identify radiation-induced responses of tissue in a label-free and non-destructive manner^23^,^24^. RS has been used previously to study the effect of proton irradiation on mouse, swine, human tumour model xenograft and human basal cell carcinoma^25^, and irradiated and unirradiated mouse brain tissue^26^. Vidyasagar *et al*., demonstrated that RS and unsupervised principal component analysis (PCA) on first derivative spectra could discriminate between responding and non-responding cervical cancer tissues collected post RT^27^. More recently, Yasser *et al*., used RS to show spectral differences in radioresistant oral cancer sublines^16^. These studies demonstrate the potential of RS to predict radioresistance and possible application of RS as a prognostic tool in cancer care and treatment.

Raman spectroscopy has been used to elucidate a radiation response in human epithelial tumour cell lines *in vitro* (NSCLC, breast, prostate), following exposure to single fractions (15, 30 or 50 Gy) of 6 MV photons^17^,^18^. These studies also showed that RS was able to separate radiation-induced spectral changes from other simultaneously occurring sources of spectral variability, such as cell cycle. In a more recent study^19^, human epithelial tumour cell lines irradiated with clinical doses of ionizing radiation (2–10 Gy) were analyzed *in vitro* (NSCLC, breast, prostate) using RS. This study further elucidated significant Raman biomarkers that could potentially be used to predict radioresistance in human tumour cells, including a dramatic increase in glycogen-related Raman spectral features following exposure to a dose of 2 Gy *in vitro* for the H460 NSCLC cell line. This response was validated using biochemical assays to show the accumulation of glycogen post-radiation, suggesting that glycogen may serve as a potential biomarker for radioresistance in human tumour cells studied *in vitro*^28^.

Unlike *in vitro* models, the tumour tissue microenvironment is a complex population of cell types (including lymphocytes, erythrocytes, stromal cells, fibroblasts and signalling molecules) as well as supporting tissue framework such as blood vasculature and extracellular matrix (ECM). This cellular heterogeneity has major consequences on tumour cell growth and progression as well as tumour response to cancer therapy, such as RT. Moreover, the metabolic constraints of low oxygen tension (hypoxia)^29^,^30^ has a classical role in radioresistance. Recently there has also been evidence to suggest the tumour-immune microenvironment has an effect on response to radiotherapy^31^, leading to immune response modification in order to enhance the efficacy of RT in cancer therapy. In the current study, we demonstrate the value of RS for identifying radiation responses even in the presence of the complex tumour microenvironment and its associated effects on radiation response. This was achieved by studying NSCLC tumour xenografts exposed to ionizing radiation, using RS in combination with PCA. This study represents the novel application of RS to identify biochemical signatures of radiation response to clinical doses of ionizing radiation in human NSCLC tumour xenografts irradiated *in vivo*, warranting further investigation of primary tissue specimens from patients receiving RT and providing new opportunities for personalizing treatment.

## Results

### Principal components and score trends with radiation exposure.

Mice were implanted subcutaneously with 5 × 10^6^ H460 cells, a human derived NSCLC model. When tumours reached 10–12 mm a 6 MV photon beam was used to irradiate the tumours *in vivo*, to doses of 0, 5 or 15 Gy. Tumours were harvested three days post-irradiation and Raman spectral maps were collected and analyzed using PCA. Principal component analysis is a widely-used multivariate analysis technique that can discriminate Raman spectra originating from biological systems^17^,^21^,^27^,^32^,^33^. All three dose groups were simultaneously analyzed using PCA, in order to reduce the spectral data set to a smaller number of variables (principal components (PCs)) that describe the majority of the variance in the spectral data set. Therefore, PCs with high contribution to the variance in the data set indicate spectral features that significantly influence the separation of spectra among the different dose groups. In this study, 90% of the total variance in the data set can be described by considering the first five PCs.

Principal components that exhibit PC score trends related to radiation exposure were identified using the chi-square statistic of a Kruskal-Wallis test. This statistic uses the PC scores labeled with their associated dose group to characterize the ratio of inter-group variability to intra-group variability. The largest chi-square values among the first five PCs were attributed to PC1 (670) and PC2 (574), with the next largest chi-square value being over 70% smaller. The chi-square values of PC1 and 2 indicate considerable inter-group variability, suggesting the variability described by these PCs can be linked to radiation exposure. The PCs represent differences between spectra with varying PC score, as previously demonstrated^19^. Due to the overlapping nature of Raman bands originating from different biomolecular entities in the spectra of biological cells and tissue, particular care has been adopted in interpretation of single peaks within a PC. Single spectral bands in a PC are only assigned to a specific chemical entity when other bands associated with that entity are also present on the same side (positive or negative) of the PC.

**Figure 1:**
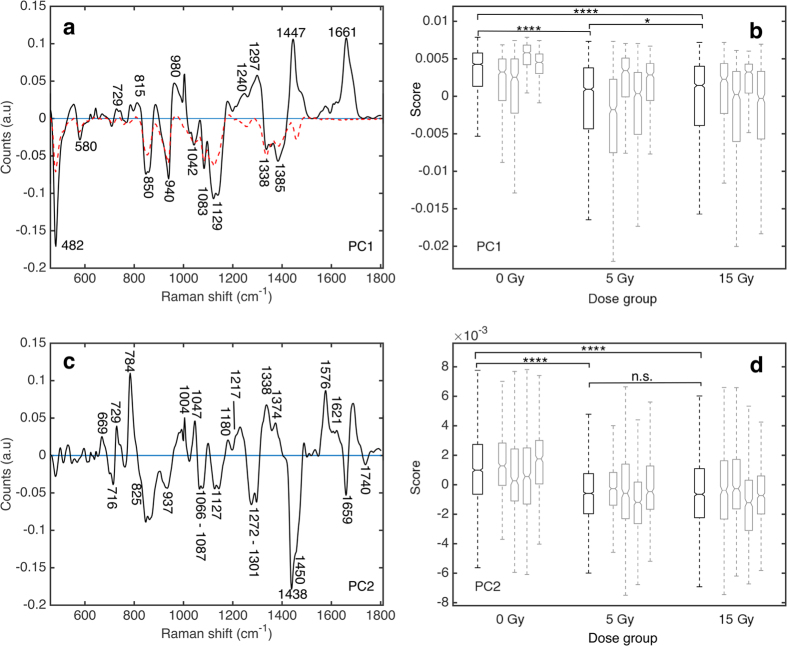
Radiation related Raman spectral changes identified using principal component analysis. Principal components (PC) 1 (**a**) and 2 (**c**) from H460 tumour tissue and corresponding PC score box plots (Tukey style box plot, outliers left out for clarity, notches indicate 95% confidence interval for the median) for PC1 (**b**) and PC2 (**d**). A statistically significant shift to more negative median PC1 and PC2 score was found for irradiated tumours compared to unirradiated tumours. Dashed red line in (**a**) corresponds to the Raman spectrum of pure glycogen (inverted, sample obtained from Life Technologies Inc., Burlington, ON, Canada). Black boxes represent PC score for all spectra collected over four tumours in a single dose group (15 Gy; n = 1969, 5 Gy; n = 2279, 0 Gy; n = 2032). Grey boxes represent PC score for all spectra collected for an individual tumour. Statistical significance was tested using a two-sided Wilcoxon rank sum test to 5% significance level, ****p-value ≤ 0.0001, *p-value ≤ 0.05, n.s. not significant.

The first principal component (PC1) isolated from the Raman data set (Fig. 1a) contributes to 69.9% of the total variance in the data set. Detailed Raman band assignment for PC1 can be found in Supplementary Table S1, indicating positive features arising from proteins and lipids and negative features from glycogen. In particular, strong positive features at 1661 cm^−1^, 1447 cm^−1^ and from 1240–1297 cm^−1^ can be linked to Amide III (random coil, β sheet and α helix) and Amide I α helix contributions in proteins, overlapping carbon-hydrogen (CH) vibrations in protein and lipids and carbon-carbon (CC) vibrational modes in lipids^34^. Negative features in PC1 at 482 cm^−1^, 850 cm^−1^, 940 cm^−1^, 1042 cm^−1^, 1083 cm^−1^, 1129 cm^−1^ and 1385 cm^−1^ can be attributed to glycogen.

Median PC score for a dose group was determined by evaluating the median PC score of spectra collected from all tumours exposed to a specific dose. Median PC1 scores from the 5 and 15 Gy dose groups (Fig. 1b) show a statistically significant shift to more negative value relative to median PC1 score for unirradiated control tumours (p-value from Wilcoxon rank sum test of 9 × 10^−122^ for 0 Gy vs. 5 Gy dose group and 1 × 10^−100^ for 0 Gy vs. 15 Gy dose group). Median PC1 scores were found to be only slightly different between the 5 and 15 Gy dose groups (p-value of 0.045). This translates to an increase in the representation of negative features in PC1 for irradiated tumours compared to unirradiated controls. The proportion of spectra with negative PC1 scores is greater for irradiated (44% for 5 Gy, 41% for 15 Gy) tumours compared to the unirradiated controls (20% for 0 Gy). Furthermore, the PC1 score corresponding to the 25^th^ percentile is −4 × 10^−3^ for the 5 and 15 Gy dose groups and 1 × 10^−3^ for the 0 Gy dose group. Therefore, 25% of the spectra comprising the 0 Gy dose group have PC1 scores more negative than 1 × 10^−3^, while in the 5 and 15 Gy dose groups 25% of the spectra have PC1 scores more negative than −4 × 10^−3^. Taken together, these data indicate that there are more spectra with strong representation of the negative features outlined in PC1, in the irradiated tumour groups compared to the unirradiated controls. This tendency towards more negative PC scores corresponds to an increase in glycogen (negative features of PC1) relative to Amide III and I protein contributions and CH and CC vibrational modes in lipids (positive features) for irradiated compared to unirradiated tumours.

The second PC (PC2) isolated from the Raman data is shown in Fig. 1c and contributes to 9.8% of the total variance in the data set. Detailed Raman band assignment for PC2 can be found in Supplementary Table S2, indicating positive features associated with nucleic acids, tryptophan and phenylalanine and negative features associated with overlapping lipid and protein bands. In particular, strong positive bands are observed at 729 cm^−1^, 1210 cm^−1^, 1338 cm^−1^, 1374 cm^−1^, 1576 cm^−1^, 1621 cm^−1^ (tryptophan), 1004 cm^−1^, 1047 cm^−1^, 1180 cm^−1^, 1210 cm^−1^ (phenylalanine), 669 cm^−1^, 1328 cm^−1^, 1342 cm^−1^, 1374 cm^−1^, 1576 cm^−1^ (guanine), 729 cm^−1^, 1342 cm^−1^, 1374 cm^−1^, 1576 cm^−1^ (adenine) and 669 cm^−1^, 784 cm^−1^, 1365 cm^−1^ (thymine)^21^,^25^,^34^,^35^. Negative bands at 1066 cm^−1^, 1080 cm^−1^, 1127 cm^−1^, 1284 cm^−1^, 1450 cm^−1^, 1460 cm^−1^, 1659 cm^−1^ indicate overlapping protein (carbon-nitrogen (CN), CH and Amide III/I α helix) and lipid (CC and CH) vibrational modes, with additional negative features at 716 cm^−1^ and 825 cm^−1^ indicating choline, as well as lipid bands at 1087 cm^−1^, 1272 cm^−1^, 1301 cm^−1^ and 1740 cm^−1^ 21,22,34.

Median PC2 scores over all spectra collected within a dose group (Fig. 1d) also show a statistically significant shift to increasingly negative PC scores for irradiated tumours compared to unirradiated controls (p-value from Wilcoxon rank sum test of 5 × 10^−109^ for 0 Gy vs. 5 Gy dose group and 7 × 10^−83^ for 0 Gy vs. 15 Gy dose group). Median PC scores were not found to be significantly different between the 5 and 15 Gy dose groups (p-value of 0.65). Furthermore, the proportion of spectra with negative PC2 scores is greater in irradiated dose groups (63% for 5 Gy and 60% for 15 Gy) compared to unirradiated controls (34% for the 0 Gy). The PC2 score corresponding to the 25^th^ percentile is −2 × 10^−3^ for the 5 and 15 Gy dose groups and −6 × 10^−4^ for the 0 Gy dose group. Taken together, the irradiated dose groups contain a larger proportion of spectra with negative PC2 scores, and stronger expression of the negative features making up PC2, compared to the unirradiated controls. This suggests an increase in negative features attributed to choline and lipid (CC, CH)/protein (CN, CH, α helix) relative to positive features attributed to nucleotides, phenylalanine and tryptophan for irradiated tumours compared to unirradiated control tumours.

Principal components 3–5 showed no considerable trends in PC score with respect to radiation exposure. Several sources of variability, such as tissue thickness and composition could manifest as contributing factors for these PCs. In summary, PCA indicates a radiation induced increase in glycogen relative to protein and lipid bands in PC1. Analysis also indicates an increase in lipid (including choline, CH, CC) and protein (CN, CH, α helix) vibrational modes relative to nucleotides, phenylalanine and tryptophan in PC2.

### Radiation-induced Raman differences in tissues correlate with *in vitro* signatures

**Figure 2:**
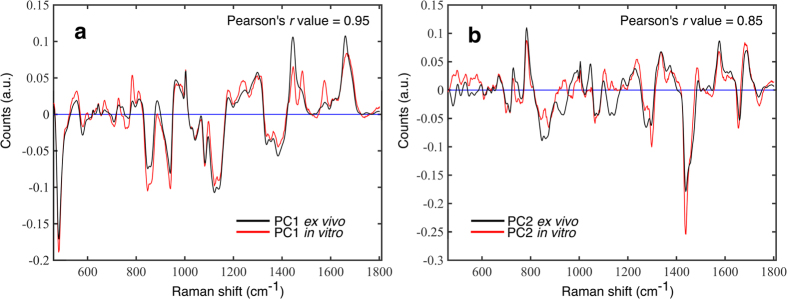
Comparison of principal components derived from Raman spectra of non-small cell lung cancer irradiated *in vitro* and *in vivo*. Principal component (PC) 1 (**a**) and PC2 (**b**) obtained for the current *ex vivo* study (black line) overlayed with the corresponding component obtained in a previous *in vitro* study on the H460 cell line. *In vitro* data was derived from principal component analysis on a Raman data set of H460 cells cultured an irradiated *in vitro* to doses of 0, 2, 4, 6, 8, 10, 30 and 50 Gy as presented and described previously^19^. Principal component 1 from this *ex vivo* study is highly correlated (Pearson’s r value = 0.95) to PC1 from the *in vitro* experiment, as is PC2 (Pearson’s r value = 0.85).

Figure 2 presents a comparison between the radiation related PCs identified in this *ex vivo* study and similar components identified in a previously published *in vitro* study on the H460 cell line (exposed to 2–50 Gy radiation doses). As shown in Fig. 2a, there is a strong correlation between the first PC identified in the *in vitro* study^19^ and the first PC identified in this *ex vivo* study (Pearson’s *r* value = 0.95). This suggests that the observed increase in glycogen content in irradiated tumours relative to unirradiated controls is consistent with *in vitro* studies using the H460 cell line. We also found a similar correlation between the second PC identified when compared to the *in vitro* study of H460 cells exposed to ionizing radiation^19^ (Pearson’s *r* value = 0.85), as shown in Fig. 2b.

### Radiation-induced Raman signatures are expressed heterogeneously throughout tissue

**Figure 3:**
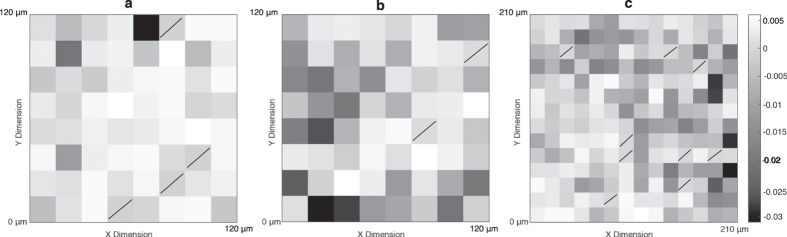
Spatial maps of principal component (PC) 1 score at three unique regions within a single tumour irradiated to 15 Gy. The spatial variation of PC1 scores demonstrates tissue heterogeneity within an individual tumour. Map areas are 120 μm^2^ for (**a**) and (**b**), 210 μm^2^ for (**c**) and images are formed with 15 μm^2^ pixels which correspond with the x and y step sizes during Raman map acquisition. Pixels with slashes indicate pixels for which no PC score information is available because the spectrum at that pixel was excluded from PCA (for reasons discussed in methods section).

Principal component 1 score was plotted as a function of position for three unique regions of a tumour exposed to 15 Gy (Fig. 3). Dark shaded regions indicate areas for which the Raman spectrum scored a relatively negative PC1 score, corresponding to a spectrum containing strong glycogen Raman bands. These maps demonstrate the diversity of PC1 score for the spectra collected within a tumour, and indicates heterogeneous spatial distribution of glycogen rich spectra within the tumour. The spread in PC score within a given tumour is a reflection of biochemical heterogeneity. During spectral acquisition, regions of visibly uneven tissue or necrotic areas within the section were avoided and maps were collected from select viable tumour regions. Therefore, despite visual consistency, large variations in biomolecular content appear to still be present. The observed intra-tumour heterogeneity in PC score is an indication that it is important to collect spectral maps from several locations within the tumour in order to avoid biasing the Raman spectral analysis of the sample.

### Expression of radiation-induced Raman signatures varies among tumours

The median PC score for each tumour (each mouse) was calculated by determining the median PC score over all spectra collected from a single tumour, and is shown in Fig. 1b,d. Among the four tumours studied in all three dose groups, median PC score was found to differ within the confidence interval on the median. For example, within the 0 Gy tumour group, median PC1 score for each of the four tumours (grey boxes, Fig. 1b) was found to be 5.8±0.2 × 10^−3^, 4.5±0.2 × 10^−3^, 3.2±0.3 × 10^−3^ and 2.5±0.5 × 10^−3^. A two-sided Wilcoxon rank sum test (p-values <0.05) identified that PC1 score distributions associated with these four tumours segregate into three unique distributions. A similar analysis found that the four tumours belonging to the 15 Gy dose group segregate into three unique sub-groups, and in the 5 Gy dose group all four tumours had unique PC1 score distributions. This suggests there is a degree of variability in the expression of PC1 across different tumours. Two out of four tumours in each of the 5 and 15 Gy dose groups have median PC1 scores that overlap with median PC1 scores of a tumour in the 0 Gy dose group (within confidence interval on the median). Therefore, some tumours express the glycogen-related RS signature (PC1) more than others, highlighting the individual variability in terms of extent of expression of this signature.

Similar grouping of PC2 score distributions among tumours (grey boxes, Fig. 1d) within a dose group was also observed. In this case, two unique tumour sub-groups formed per dose. Within confidence interval on the median, there was no overlap of the median PC scores between irradiated and unirradiated dose groups for PC2. This data further supports the observation that the extent of radiation-induced Raman spectral changes may be unique to an individual.

### Glycogen accumulation confirmed using Periodic acid-Schiff staining

**Figure 4:**
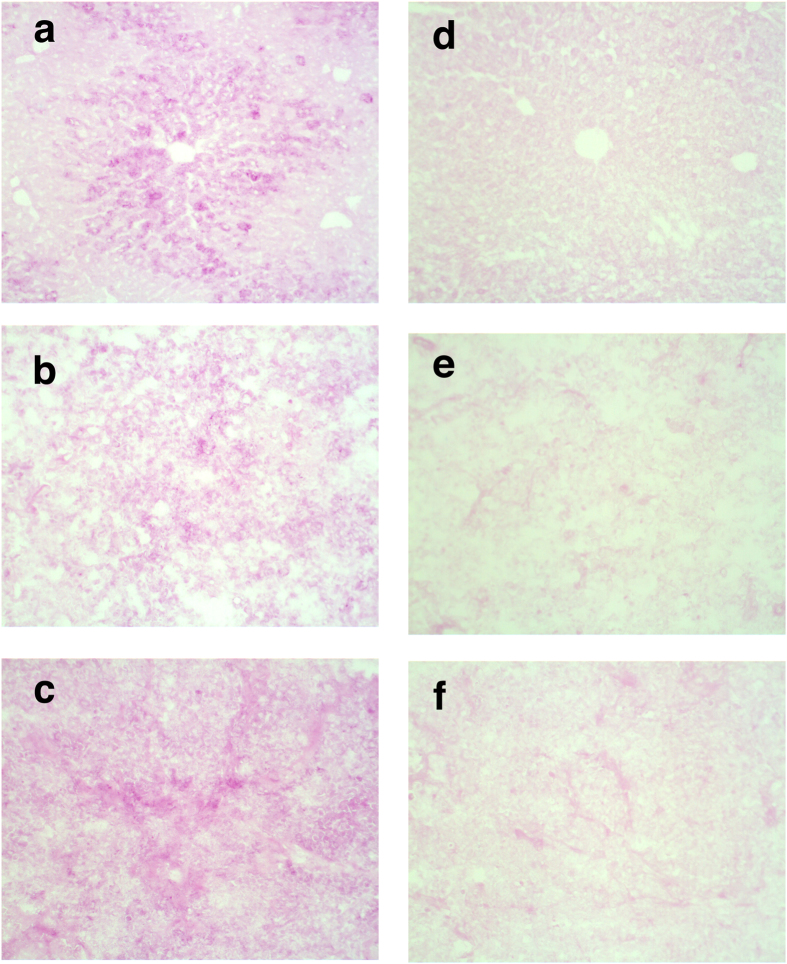
Periodic acid–Schiff (PAS) staining images from irradiated and unirradiated tumour tissue and liver tissue control. A qualitative indication of increased glycogen content in irradiated (15 Gy) tissue compared to unirradiated (0 Gy) tissue was given by PAS staining. Images are collected at 20× magnification. PAS stained (**a**) Liver tissue (positive control) without diastase (alpha-amylase) and (**d**) with diastase. (**b**) Unirradiated control (0 Gy) H460 tumour tissue without diastase (alpha-amylase) and (**e**) with diastase. (**c**) Irradiated (15 Gy) H460 tumour tissue without diastase (alpha-amylase) and (**f**) with diastase.

To confirm the observed accumulation of glycogen in irradiated tumours, as indicated through PC1 score trends, we performed a periodic acid-Schiff (PAS) stain to visualize the total intracellular content of glycogen of tumour fragments that were harvested at three days post-irradiation. Normal liver tissue contains a large amount of glycogen and was used as a positive control. Intense PAS staining was observed in tissue sections of unirradiated control hepatocytes (Fig. 4a). A weak but detectable PAS-staining of glycogen granules from tissue sections of an unirradiated tumour is shown in Fig. 4b. In contrast to this, tissue sections from the 15 Gy irradiated tumour showed a strong PAS staining of glycogen granules (Fig. 4c).

Diastase (alpha-amylase) was used to selectively digest glycogen prior to PAS staining (Fig. 4d–f). In all tissues examined, diastase reduced the intensity of PAS staining but did not fully eliminate it. This is due to background staining of other tissue constituents such as mucin, glycated proteins and basement membranes. Qualitatively, the difference in degree of PAS staining between digested unirradiated and irradiated tissue is less drastic than between undigested unirradiated and irradiated tissue, providing additional support for the observed accumulation of glycogen as a metabolic response to irradiation, found using RS. Although there are disadvantages related to the specificity of the PAS stain, the distribution of PAS stained regions throughout the tissue further highlights the intratumoural spatial variability in PC1 score in Raman spectra measured throughout a given tumour.

## Discussion

We have demonstrated the utility of RS in combination with PCA to detect radiation-induced biochemical changes in human NSCLC tumour (H460) xenografts. Analysis revealed unique radiation-related Raman signatures that can be linked to four major groups of biomolecules: nucleic acid, lipid, protein and carbohydrates.

The influence of radiation on nucleotide content was observed in PC2 through a radiation-induced reduction in Raman bands associated with guanine, adenine and thymine relative to protein and lipid spectral features. Principal component 1 did not contain peaks that could yield to an interpretable change in nucleic acid content.

Radiation-induced effects on lipid content were identified in PC1 as a reduction of CC and CH vibrational modes relative to glycogen. Furthermore, PC2 indicated a radiation-related increase in Raman bands associated with choline, CC, CH, and ester vibrational modes in lipids relative to nucleotide and protein bands associated with phenylalanine and tryptophan.

Analysis revealed radiation-induced effects on protein content in PC1 as a reduction in Amide III (random coil, α helix and β sheet) and Amide I α helix bands relative to glycogen. Furthermore, PC2 identified a radiation-induced reduction in tryptophan and phenylalanine Raman bands. The second component also identified a radiation-induced increase in bands associated with CN and CH protein vibrational modes as well as bands associated with α helix protein conformation, relative to features associated with nucleotides, tryptophan and phenylalanine.

Radiation-induced effects on carbohydrates were observed through PC1, which indicated an increase in glycogen spectral features in 5 and 15 Gy populations compared to unirradiated tumours. The second PC did not contain peaks that provided an interpretable change in carbohydrates within the tissue. The variety of radiation induced spectral changes observed over nucleic acid, lipid, protein and carbohydrate content in this study indicate that multiple physiological or metabolic processes may be occurring within the tissue, in response to radiation.

Previous *in vitro* studies have linked the features in PC2 to variation in cellular nucleic acid, lipid and protein content as a result of variations in progression through the cell cycle^36^. However, unlike in this *ex vivo* experiment, segregation in PC score between irradiated and unirradiated populations was not observed *in vitro*. The segregation in PC2 score between irradiated and unirradiated populations may suggest a certain degree of cell cycle synchronization in the *ex vivo* case, however further studies are needed to assert this hypothesis.

The radiation-induced increase in glycogen containing spectra identified in irradiated tissue in this study, is consistent with *in vitro* studies carried out on the H460 cell line^19^,^28^ and was confirmed qualitatively using PAS stain. Furthermore, an earlier study reported a glycogen accumulation in brain tissue exposed to ionizing radiation^37^, supporting our observations in this study. While the mechanisms for radiation-induced glycogen accumulation are not fully understood *in vitro*, a radiation-induced inactivation of GSK-3β has been speculated to contribute to an increased capacity of glycogen synthase to produce cellular glycogen^28^. However, *in vivo* tumours comprise a significantly more complex system characterized by a heterogeneous microenvironment with an anisotropic distribution of hypoxic, glucose deprived and high lactate regions^6^. It is possible that the complex tumour physiology and biochemistry contributes to the mechanisms that yield the observed glycogen accumulation post RT. Specifically, hypoxic cells are more radioresistant than oxic cells^38^,^39^, causing oxic cells to be preferentially killed during RT. This leads to redistribution of blood within the tumour allowing for a degree of hypoxic recovery in irradiated compared to unirradiated tumours^40^. Glycogen accumulation has been shown to occur in brain tissue recovering from hypoxia^41^, therefore this may be a possible explanation for increased expression of glycogen Raman spectral features in irradiated tumours.

The observed intra-tumour heterogeneities as well as the inter-individual variability in degree of expression of the glycogen Raman signature support the hypothesized link between increased glycogen post RT and hypoxia/hypoxic recovery. The tumour microenvironment is a complex mixture of different cell types and is characterized by drastic differences in pH, oxygen concentrations and nutrient distributions^30^. Intra-tumour heterogeneities in expression of the glycogen-related Raman signature may be a result of this inhomogeneity in microenvironment. Genetics and proteomics has unveiled crucial intra-tumour heterogeneities indicating evolutionary and protein function distributions which may affect prognostic evaluations of the disease^42^,^43^. Here, using Raman spectroscopic imaging, we uncover glycogen as a previously under-appreciated factor to define a metabolic signature that may delineate intra-tumoural heterogeneity in NSCLC.

Tumour development (for example: vasculature development, cellular growth kinetics) plays an intrinsic role in determining the microenvironment of a tumour, including location and extent of regions of hypoxia throughout the tumour^6^, and studies have shown that the degree of hypoxia can vary substantially among patients^38^. Since inbred mice are used as hosts and the tumour cells used in each mouse originate from the same source, inter-tumour variability may be linked to differences in microenvironment between tumours. Variations in the extent of hypoxia among individual mice and thus extent of hypoxic recovery following radiation exposure may therefore be an explanation for the variability in measured levels of glycogen for tumours exposed to the same dose of ionizing radiation. The observed inter-individual variability is further evidence of the unique radiation response associated with individuals based on their unique disease, and substantiates the notion of characterizing an individual’s degree of radioresistance or sensitivity and personalizing RT because the metabolic response to radiation exposure is not the same for every individual.

This study represents the novel application of RS to identify biochemical signatures of radiation response in human NSCLC tumour xenografts, irradiated *in vivo*. Multiple physiological or metabolic processes were measured within the tissue, in response to radiation, including a marked increase in glycogen spectral features in irradiated tumours. Spatial mapping revealed both intra- and inter-tumour heterogeneity in the distribution of glycogen and other RS spectral features. Collectively, these data demonstrate the utility of RS for detecting distinct radiobiological responses in human tumour xenografts, indicating the potential for future RS applications to monitor or predict radiation response in individuals.

## Methods

### Cell lines and tumour xenografts

All animal procedures were approved by the University of Victoria Animal Care Committee and were performed in accordance with the Canadian Council on Animal Care. 6–8 week-old NOD.CB17-Prkdcscid/J female mice were obtained from British Columbia Cancer Research Center (BCCRC) Animal Resource Center (Vancouver, BC). Animals were housed in microisolater cages, and allowed access to food and water *ad libitum*. Animals were acclimatized for 1 week prior to study initiation.

The animals were anesthetised by isoflurane inhalation (1–3% for maintenance; up to 5% for induction) in oxygen from a precision vaporizer. The fur from the flank of animals was shaved before tumour cell implantations. The human NSCLC cell line H460 (purchased from American Type Culture Collection, Manassas, VA, USA, ATCC# HTB-177, passage# 4, not listed as misidentified, tested pathogen-free with Charles River Laboratories, Montreal, QC, Canada) at a concentration of 5 × 10^6^ cells in 0.1 ml PBS, were injected subcutaneously in the right flank of each animal.

### Tumour irradiation and harvesting

Pre-determined exclusion criteria disqualified animals from receiving radiation treatment and continuing on in the study if the tumour was above 12 mm or below 10 mm. Tumours were measured three times per week with digital calipers and animals were randomized into treatment groups once their tumour size reached the pre-determined acceptable tumour size. To reduce any possible nosocomial infections during irradiation, a broad spectrum antibiotic (Enrofloxacin) was added to the drinking water 10 days prior to RT and the mice remained on this prophylaxis treatment until the end of the study. No blinding of the investigators to radiation dose received by a given mouse was done.

Once tumour sizes reached 10–12 mm diameter, the mice were prepared for tumour irradiation. Each mouse was anaesthetized using a single intraperitoneal (IP) injection of Ketamine/Dexdomitor mixture, dosed appropriately for body weight using 50 mg/kg Ketamine and 0.5 mg/kg Dexdomitor. After confirmation of anaesthesia using a firm toe pinch, individual mice were placed into a custom-designed restraining acrylic chamber and a 10 mm non-toxic removable dental wax bolus was placed over the irradiation area. This chamber was placed near the isocentre of a Varian Truebeam STx linear accelerator (LINAC) (Varian Medical Systems Inc., Palo Alto, CA, USA). For radiation delivery, treatment dose plans utilizing parallel-opposed beam geometries were developed for tumour diameters of 10–15 mm using a phantom mouse and Eclipse (version 11) treatment planning software (Varian Medical Systems Inc.). The posterior base of the mouse tumour was aligned with the treatment isocentre using the On-Board Imager® (OBI) feature of the Varian Truebeam linear accelerator. Irradiations were carried out using a 6 MV photon beam with a nominal dose rate of 6 Gy/minute at isocentre. Depending on the treatment group, single fractions of 0, 5 and 15 Gy were delivered to the tumour tissue. Following irradiation, the mice were given 1 mg/kg Atipamezole sedation reversal IP and mice were allowed to recover with thermal support.

Three days post-irradiation, mice were euthanized and tumours were removed. Tissue for Raman spectral analysis was embedded in mounting medium (Tissue-Tek O.C.T.™ Sakura Finetek Europe B.V., The Netherlands), snap frozen in liquid nitrogen, and stored at −80 °C.

### Raman microscopy measurements of tumour tissue

Tumours were prepared into 20 μm sections using a rotary microtome (HM 550; MICROM International GmbH, Walldorf, Germany) and placed on magnesium fluoride slides. Just prior to Raman measurements, these frozen sections were air-dried for 10 minutes. A Renishaw inVia Raman Microscope (Renishaw Inc., Illinois, IL, USA) coupled to a 785 nm diode laser (Renishaw) and dry objective (100x, NA = 0.9) (Leica Microsystems, Wetzlar, Germany) was used to collect Raman map spectra. Spectra were registered using a thermoelectrically cooled charge coupled device (CCD) detector (Andor Technology, Connecticut, USA). The laser sampling dimensions were 2 × 5 × 10 μm^3^ and a laser power density at the sampling volume was 0.5 mW/μm^3^. Spectra were acquired for 20 seconds per point, covering a spectral range of 460–1800 cm^−1^.

A total of twelve mice were studied, with four mice in each dose group (0, 5 and 15 Gy). Animal sample size was selected to follow similar population sizes from previously published Raman studies involving animals and radiation exposure^26^. Five to eight unique mapping regions (map areas are between 100–220 μm^2^, step size 15 μm) were analyzed over three tissue sections per mouse; resulting in a total of 6648 spectra prior to spectral processing. This experiment was repeated twice in the lab, however representative results from one of the studies is presented.

### Spectral processing and statistical analysis

Spectra containing cosmic rays and obvious outliers (eg. saturated spectra) were manually identified and removed from the data set. Following outlier removal, the data set contained a total of 6280 spectra. In-house algorithms were used to estimate and subtract spectral baseline arising from the substrate and biological fluorescence, shift spectra to account for calibration drifts during data acquisition, and normalize spectra to the total amount of biological material within the sampling volume, as previously outlined^36^.

Baseline removal was carried out independently for each spectrum, using a modified signal removal method, tested and described in detail elsewhere^44^. Briefly, the baseline of a spectrum is initially estimated using a Savitzky-Golay zero-order filter. Spectral points with values above the estimated baseline are identified and subsequently reduced by a pre-specified amount to yield a residual spectrum. This residual spectrum is used to produce a new estimate of the baseline using the Savitzky-Golay filter and the process is reiterated over a pre-determined number of iterations. Following baseline removal, individual spectra were normalized to the total area under the baseline corrected spectrum.

Spectral shifting was carried out using the phenylalanine peak located at 1003 cm^−1^, as described in detail elsewhere^45^. Briefly, the central wavenumber of the phenylalanine peak is identified for each spectrum using a Gaussian fit. The phenylalanine peak location in the spectrum to be shifted is compared to the peak location of the reference spectrum (difference was always less than 1 cm^−1^) and a point-by-point interpolation of the spectrum is used to shift the spectrum by the required amount. This process is iterated twice, resulting in alignment of the phenylalanine peaks for the reference spectrum and shifted spectrum.

Principal component analysis, was used to separate out those components of the spectral dataset which contain variability due to radiation exposure and those contributed from other sources. Tukey style box plots, showing median PC score and 25^th^ and 75^th^ percentiles are used to represent PCA results for a given tumour and for a dose group (data set is comprised of all useable processed spectra from all tumours receiving the same dose). The most extreme data points, defined by the PC score closest to 1.5*(75^th^ percentile-25^th^ percentile) are represented by the whiskers in the box plots, and outliers (PC scores greater than the whiskers) are left off of the plots to improve clarity. Notches indicate the 95% confidence interval on the median, approximated assuming a normal distribution, but is accurate for large samples of other distributions as well^46^. The 0 Gy dose group has a sample size of n = 2032 spectral points (n = 436, 605, 557, 434 for each of the tumours), 5 Gy dose group n = 2279 (n = 755, 681, 437, 406 for each of the tumours) spectral points, 15 Gy dose group n = 1969 (n = 557, 552, 432, 428 for each of the tumours) spectral points.

Statistically significant shifts in PC score over a single tumour as well as over the entire dose group was tested using a two-sided Wilcoxon rank sum test to a 5% significance level. The chi-square statistic of a Kruskal-Wallis test was used to estimate the relevance of the PCs in describing variability related to radiation dose (a non-parametric replacement for the f-statistic from a one-way ANOVA). The Kruskal-Wallis test is an extension of the Wilcoxon rank sum test beyond two groups. Both tests assume similar standard deviation among the test populations, a condition that is satisfied by the test populations examined. Comparison between *in vitro* and *in vivo* experiments was done using a Pearson’s *r* value calculation.

### Periodic acid–Schiff staining

Periodic acid–Schiff staining was performed using PAS reagent kit (Cat # 395-1; Sigma Aldrich, St. Louis, MO, USA). In this study, tumour tissue from a mouse irradiated with 15 Gy and unirradiated control tumour are presented. Mouse liver tissue was also studied using PAS staining, to act as a positive control for glycogen. Frozen tumour tissue was cut into 10 μm sections using a Rotary Microtome (HM 355 S; MICROM International GmbH, Walldorf, Germany) and adhered directly to a glass slide. Slides were fixed in 10% buffered formalin for 30 minutes at room temperature, washed three times in PBS, then rinsed in deionized (DI) water. Samples were then exposed to 1% periodic acid at room temperature for 5 minutes followed by three washes in DI water. Samples were stained with Schiff reagent for 15 minutes at room temperature, followed by a wash under running water for 10 minutes and a quick rinse in DI water. Samples were then dehydrated in 95% ethanol for 1 minute followed by three sequential submersions of the tissue in 100% ethanol. Eco-Mount mounting medium (Biocare Medical, Concord, CA, USA) was used for slides and imaged under bright field microscope at 20× magnification. Diastase (alpha-amylase) digestion (0.5%) was used to remove glycogen from samples providing a negative control.

### Code Availability

All computer code (to carry out spectral processing, data analysis, statistical testing) was derived from standard algorithms in Matlab (Mathworks, Natick, MA, USA), version R2014B, and can be accessed through this program.

## Additional Information

**How to cite this article**: Harder, S. J. *et al*. Raman spectroscopy identifies radiation response in human non-small cell lung cancer xenografts. *Sci. Rep.* **6**, 21006; doi: 10.1038/srep21006 (2016).

## Supplementary Material

Supplementary Information
