# Supplementary material for: Raman spectroscopy identifies radiation response in human non-small cell lung cancer xenografts
Source: Sci Rep. 2016 Feb 17;6:21006. doi: 10.1038/srep21006 (PMC4756358; doi:10.1038/srep21006)
Supplement: Supplementary Information [file srep21006-s1.pdf]

Supplemental material

Supplementary Table S.1: Raman peak assignment for radiation induced principal component 1 (PC1). Only main spectral contributors to the PC are listed. Italicised assignments indicate negative PC features.

	Assignment		
Raman Shift (cm^{-1})	Protein	Lipid	Carbohydrate
482			*glyc.*
580			*glyc.*
729		C-N h.g.[1]	
815		O-P-O[1]	
850			*glyc.*
940			*glyc.*
980		C-C h.g.[1]	
1042			*glyc.*
1083			*glyc.*
1129			*glyc.*
1240	Amide III r.c.[1]		
1245	Amide III β sheet[1]		
1274	Amide III β sheet[1]	=CH def.[1]	
1284	Amide III α helix[1]	=CH def.[1]	
1297		CH_2 tw.[1]	
1338			*glyc.*
1385			*glyc.*

	**Assignment**		
**Raman Shift (cm^{-1})**	**Protein**	**Lipid**	**Carbohydrate**
1447	CH def.[1]	CH def.[1]	
1465	CH def.[1]	CH def.[1]	
1661	Amide I α helix[1]	C=C str.[1]	

glyc. - glycogen, h.g. - head groups, r.c. – random coil, def. - deformation, tw. - twist, str. - stretch.

Supplementary Table S.2: Raman peak assignment for principal component 2 (PC2). Only main spectral contributors to the PC are listed. Italicised assignments indicate negative PC features.

	**Assignment**		
**Raman Shift (cm^{-1})**	**Nucleic Acid**	**Protein**	**Lipid**
669	G, T[2]		
716			*Cho.*[2]
729	A[1]	Trp.[1]	
784	T[1]		
825			*Cho.*[3]
937		*C-C bk. str. α helix*[1]	
1004		Phe.[1]	
1047		Phe.[2]	
1066		*C-N str.*[1]	*Chain C-C str.*[1]
1080		*C-N str.*[1]	*Chain C-C str.*[1]
1087			*Chain C-C str.*[1]
1127		*C-N str.*[1]	*Chain C-C str.*[1]
1180		Phe.[2]	
1210		Phe., Trp.[1]	
1272			*=CH def.*[2]
1284		*Amide III α helix*[1]	*=CH def.*[1]
1301			*CH_2 tw.*[1]
1328	G[1]		
1338		Trp.[4]	

	Assignment		
**Raman Shift (cm^{-1})**	**Nucleic Acid**	**Protein**	**Lipid**
1342	A, G[1]		
1365	T[2]		Sym. str. CH_3[1]
1374	A, G[5]	Trp.[4]	
1450		*CH def.*[1]	*CH def.*[1]
1460		*CH def.*[1]	*CH def.*[1]
1576	A, G[5]	Trp.[4]	
1607		Phe.[1]	
1621		Trp.[1]	
1659		*Amide I α helix*[1]	*C=C str.*[1]
1740			*>C=O ester*[1]

cho. – choline, bk. - backbone G - guanine, A - adenine, T - thymine, Phe. - phenylalanine, Trp. - tryptophan, def. – deformation, tw. – twist, sym. – symmetric, str. – stretch.

References:

1. Notingher, I., Verrier, S., Haque, S., Polak, J. M. & Hench, L. L. Spectroscopic study of human lung epithelial cells (A549) in culture: Living cells versus dead cells. *Biopolym. - Biospectroscopy Sect.* **72,** 230–240 (2003).

2. Delfino, I. *et al.* Visible micro-Raman spectroscopy of single human mammary epithelial cells exposed to x-ray radiation. *J. Biomed. Opt.* **20,** 035003 (2015).

3. Maguire, A. *et al.* Analyses of ionizing radiation effects in vitro in peripheral blood lymphocytes with Raman spectroscopy. *Radiat. Res.* **183,** (2015).

4. Synytsya, A. *et al.* Raman spectroscopy of tissue samples irradiated by protons. *Int. J. Radiat. Biol.* **80,** 581–591 (2004).

5. Yu, C., Gestl, E., Eckert, K., Allara, D. & Irudayaraj, J. Characterization of human breast epithelial cells by confocal Raman microspectroscopy. *Cancer Detect. Prev.* **30,** 515–522 (2006).
